# Combined-Acupoint Electroacupuncture Induces Better Analgesia via Activating the Endocannabinoid System in the Spinal Cord

**DOI:** 10.1155/2022/7670629

**Published:** 2022-09-15

**Authors:** Zhenhua Jiang, Yuheng Li, Qun Wang, Zongping Fang, Jiao Deng, Xinxin Zhang, Bowen Shen, Zhixin Wu, Qianzi Yang, Lize Xiong

**Affiliations:** ^1^Department of Anesthesiology and Perioperative Medicine, Xijing Hospital, Fourth Military Medical University, Xi'an, Shaanxi Province 710032, China; ^2^Department of Anesthesiology, The 960th Hospital of PLA, Jinan, China; ^3^Department of Anesthesiology and Perioperative Medicine, Translational Research Institute of Brain and Brain-Like Intelligence, Shanghai Fourth People's Hospital Affiliated to Tongji University School of Medicine, the Shanghai Key Laboratory of Anesthesiology and Functional Modulation, Shanghai 200434, China

## Abstract

Electroacupuncture (EA) therapy has been widely reported to alleviate neuropathic pain with few side effects in both clinical practice and animal studies worldwide. However, little is known about the comparison of the therapeutic efficacy among the diverse EA schemes used for neuropathic pain. The present study is aimed at investigating the therapeutic efficacy discrepancy between the single and combined-acupoint EA and to reveal the difference of mechanisms behind them. Electroacupuncture was given at both Zusanli (ST36) and Huantiao (GB30) in the combined group or ST36 alone in the single group. Paw withdrawal mechanical threshold (PWMT) was measured to determine the pain level. Electrophysiology was performed to detect the effects of EA on synaptic transmission in the spinal dorsal horn of the vGlut2-tdTomato mice. Spinal contents of endogenous opioids, endocannabinoids, and their receptors were examined. Inhibitors of CBR (cannabinoid receptor) and opioid receptors were used to study the roles of opioid and endocannabinoid system (ECS) in EA analgesia. We found that combined-acupoint acupuncture provide stronger analgesia than the single group did, and the former inhibited the synaptic transmission at the spinal level to a greater extent than later. Besides, the high-intensity stimulation at ST36 or normal stimulation at two sham acupoints did not mimic the similar efficacy of analgesia in the combined group. Acupuncture stimulation in single and combined groups both activated the endogenous opioid system. The ECS was only activated in the combined group. Naloxone totally blocked the analgesic effect of single-acupoint EA; however, it did not attenuate that of combined-acupoint EA unless coadministered with CBR antagonists. Hence, in the CCI-induced neuropathic pain model, combined-acupoint EA at ST36 and GB30 is more effective in analgesia than the single-acupoint EA at ST36. EA stimulation at GB30 alone neither provided a superior analgesic effect to EA treatment at ST36 nor altered the content of AEA, 2-AG, CB1 receptor, or CB2 receptor compared with the CCI group. Activation of the ECS is the main contributor of the better analgesia by the combined acupoint stimulation than that induced by single acupoint stimulation.

## 1. Introduction

Neuropathic pain, defined as the chronic pain condition caused by a lesion or disease of the somatosensory nervous system [1], is a serious problem threatening the health of human. The prevalence rate of neuropathic pain in general population is estimated to be as high as 8% [2, 3]. Nearly 30% of people in the United States suffer from neuropathic pain, resulting in an economic cost of 560-635 billion US dollars annually [4]. More importantly, the commonly used medications for neuropathic pain have a limited efficacy, and serious side effects are inevitable [5].

Acupuncture, which refers to stimulation of acupoints to modulate the body physiology [6, 7] and related techniques, such as electroacupuncture (EA), has been widely reported to alleviate pain in both clinical practice [8, 9] and animal studies [10–12] with few side effects. The National Institutes of Health (NIH) has clearly recommended acupuncture as an alternative therapy when conventional treatment is not satisfactory [10]. Meanwhile, increasing number of people have taken acupuncture treatment as part of medical care in management of pain. However, acupuncture schemes vary from studies and lack a standard practice. Acupoint selection determines the effect of acupuncture therapy to a large extent [13]. Although previous studies reported that stimulations at both single acupoint and multiple acupoints were effective in neuropathic pain treatment [14–18], such as single acupoint of ST36 [19, 20] or combinations of ST36 and GB30 [21, 22], very few researches compared the therapeutic efficacy among the diverse acupuncture schemes. To investigate whether there is a difference of analgesia between single- and combined-acupoint scheme is of great importance for simplifying and standardizing the practice of acupuncture.

It has been established that increase of excitability of spinal dorsal horn neurons and the synaptic strength between C fiber and spinal dorsal horn neurons contribute to neuropathic pain [23, 24]. Electroacupuncture has been proved to relieve neuropathic pain mainly at spinal level by involving endogenous opioids system, serotonin, norepinephrine, amino acids, and glia cell/cytokines [25]. Particularly, the endogenous opioid system in the spinal cord has been widely reported to participate in EA analgesia of neuropathic pain in humans and animals [10]. Besides, it has been acknowledged that low frequency (2-10 Hz) EA exerts a stronger analgesia than high frequency (100 Hz) EA in inhibiting inflammatory pain, which mediated by met-enkephalin, *β*-endorphin, and dynorphin, respectively [26], but whether this specificity applies to neuropathic pain remains unknown [25]. In addition, ECS also contributes to the EA analgesia [27]. The endocannabinoid system is composed of the endocannabinoids (anandamide, AEA; 2-arachidonoylglycerol, 2-AG), cannabinoid receptors (CB_1_R, CB_2_R), synthetase, and hydrolase of endocannabinoids [28, 29]. A preliminary study concluded that CB1R and CB2R are involved in the EA-induced analgesia and anti-inflammatory effects, respectively [27]. Although combined-acupoint EA could suppress inflammatory pain via regulating ECS, it produced analgesia in the supraspinal region. A very typical model of chronic pain is the chronic constriction injury of the sciatic nerve (CCI) in rats, which shows obvious mechanical allodynia. Whether ECS participates in the EA induced analgesia at the spinal cord is not clear. Thus, to investigate the analgesic effects and the underlying mechanism of EA treatment at single and combined acupoints may provide a structural and functional basis for developing more optimal EA strategy.

In the present study, we aim to answer the following questions: (1) Is combined-acupoint EA more effective than single-acupoint EA for analgesia in CCI-induced neuropathic pain? (2) What is the underlying analgesic mechanism of single-acupoint EA or/and combined-acupoint EA? Hence, we firstly established a chronic neuropathic pain model in rats and compared the analgesic efficacy of single-acupoint (ST36) and combined-acupoint (ST36 + GB30) EA through testing pain behaviors. In addition, we also examined the effects of single and combined EA on the pain information transmission in the spinal dorsal horn. Then, we further explored the mechanism underlying their analgesic effects using western blots, ELISA assays, and intrathecal injection methods. Elucidating the differences in EA-induced antinociception between single-acupoint and combined-acupoint schemes, as well as the underlying mechanism, may accelerate the development of drugs and provide better choices for patients that are refractory to conventional therapy.

## 2. Materials and Methods

### 2.1. Animals

Six weeks old male Sprague–Dawley rats weighing 167.8 ± 7.3 g (purchased from the Experimental Animal Center of the Fourth Military Medical University, Xi'an, Shaanxi, China) were used in the behavioral, immunohistochemistry, western blot, and ELISA experiments. Adult heterozygous male vGlut2-Cre mice (Jackson Laboratories) were crossed with Ai9 reporter mice (Jackson Laboratories) to generate vGlut2-tdTomato mice. Young adult (3–5 weeks old) male vGlut2-tdTomato mice were used for electrophysiological experiments. Animals were group-housed (4 per cage) at a temperature of 22-24°C, with a 12-hour light/dark cycle and free access to food and water. All rats were acclimatized to the laboratory conditions for at least 7 days before experimental manipulation in case their stress responses affected the experiment results. All animal experiments were approved by the Ethic Committee of the Fourth Military Medical University and followed the policies for the use of laboratory animals issued by the International Association for the Study of Pain. All efforts were made to minimize the number of animals used and their suffering.

### 2.2. Study Design

For rats, the whole study was divided into three steps. In the first step, rats were randomly divided into control, CCI, single, and combined groups (*n* = 10 per group). Sham operation was done in the control group, while the CCI model was established in the other 3 groups. After the CCI model were established, different treatments were applied to rats for 2 weeks: immobilization for the control and CCI group, ST36-acupoint EA for the single group, and ST36 + GB30-acupoint EA for the combined group. Paw withdrawal mechanical threshold of the rats, as well as the expression of endorphin, enkephalin, MOR, DOR, AEA, 2-AG, CB_1_R, and CB_2_R, were measured.

Secondly, rats were divided into CCI, single, high-intensity, combined, and sham-combined group randomly (*n* = 8 per group), and all the rats received CCI injury. Treatments in CCI, single, and combined group were the same as the first step. Single-acupoint EA with high intensity stimulation was applied to high-intensity group, and sham-combined group was stimulated at sham acupoints. Paw withdrawal mechanical threshold was then measured to evaluate the analgesic effects in each group.

Thirdly, rats were randomly grouped into CCI, single, combined, and combined + CBR (cannabinoid receptor) inhibitor group (*n* = 8 per group), and all rats were conducted with CCI surgery. Except for immobilization and EA treatment, nonselective opioid antagonist naloxone was administrated to all animals intraperitoneally; CB_1_R and CB_2_R antagonists were applied intrathecally in the combined + CBR group. Mechanical threshold was then measured.

Finally, rats were randomly divided into CCI, ST36, and GB30 groups (*n* = 8 − 10). All rats were performed with CCI surgery. After the rats were immobilized and delivered EA treatment, PWMT as well as the expression of AEA, 2-AG, CB_1_R, and CB_2_R was measured.

For vGlut2-tdTomato mice, they were divided into control, CCI, single, and combined groups (*n* = 6). Control and CCI groups received sham and CCI surgery, respectively, and they are used for electrophysiological experiment at 10 days after operation. For combined (ST36 + GB30) and single (ST36) groups, they received CCI and EA treatment started at the 10 days after CCI. After the 6-consecutive days' treatment and 1-day off, they were used for electrophysiological experiment.

### 2.3. Neuropathic Pain Model

The chronic constriction injury (CCI) model was established as previously described to investigate the analgesic effect of electroacupuncture (EA) [30, 31]. Briefly, after the rat or vGlut2-tdTomato mouse was anesthetized with 1.5% isoflurane in oxygen, the left sciatic nerve was exposed. Four 4-0 chromic gut sutures were then tied to the sciatic nerve to induce the injury, and the surgical site was then closed with silk sutures. The behavioral test was conducted at 10 days after modeling to ensure the reliability of the pain phenotype.

### 2.4. EA Treatment

Rats were gently immobilized by our homemade fixing device without anesthesia. The vGlut2-tdTomato mice were anesthetized by 3.0% isoflurane and maintained by 1.5% isoflurane. The stainless-steel acupuncture needles (0.1 mm in diameter, Huatuo, Suzhou, China) were inserted into bilateral ST36, GB30, or nonacupoints according to the grouping. The needles were connected to the Huatuo SDZ-V Nerve and Muscle Stimulator (Huatuo), and then the dense-and-disperse mode stimulation was given at 2/10 Hz. Generally, the lowest intensity (1-2 mA) of EA which could evoke the vibration of the stimulated hindlimb was chosen for each acupoint. As to the high-intensity stimulation, the maximum intensity of EA that animals could tolerate was adopted. EA was given for 30 minutes per day. An EA treatment course included 6-consecutive days' treatment and 1-day off. EA treatment started at the 10 days after CCI.

### 2.5. Pain Behavioral Test

Paw withdrawal mechanical threshold (PWMT) was measured by von Frey filaments. Rats or mice were habituated in the experimental apparatus for 30 min, and baseline of PWMT was measured. After the CCI model was constructed, the EA treatment was performed. On Wednesday, Friday, and Sunday every week, animals were firstly treated with EA followed by detecting PWMT, which lasts for 2 weeks.

During the tests, each rat or mouse was placed in a chamber (15 cm × 15 cm × 15 cm) on a platform with 5 mm grids of iron wires throughout the entire area. The up-down method was used to evaluate mechanical allodynia as we previously did [32]. Briefly, the PWMT was determined by using von Frey hairs (Stoelting, Wood Dale, USA) applied to the central region of the plantar surface of the left hind paw in ascending order (rat: 2-26 g; mice: 0.008-2 g). Each filament was tested for 10 times at 10 s intervals. The PWMT was defined as the lowest force in grams that produced at least 5 withdrawal responses in 10 consecutive applications. All the tests were conducted by a researcher who is blind to the grouping.

### 2.6. Preparation of Sagittal Lumbar Spinal Cord Slice Attached with a Dorsal Root

According to the previous study [33], sagittal lumbar spinal cord slices (400- to 500 *μ*m-thick) attached with a dorsal root were prepared. Briefly, young adult vGlut2-tdTomato mice from control, CCI, combined, and single groups were transcardially perfused with ice-cold sucrose artificial cerebrospinal fluid after deeply narcotized with pentobarbital sodium. Then, the sagittal lumbar spinal cord (400- to 500 *μ*m-thick) with dorsal root was removed and cut by a vibrating microtome filled with ice-cold sucrose cerebrospinal fluid. Finally, lumbar spinal cord slice was incubated in the normal cerebrospinal fluid equilibrated with a mixture of 95% O_2_ and 5% CO_2_ at room temperature for 1 h.

### 2.7. Patch Clamp Whole Cell Recordings

According to our previous electrophysiology protocol [33], resistance of patch pipettes was maintained at 5 to 10 M*Ω*. Tight whole cell recordings were made from vGlut2-positive neurons located in lamina I and IIo of spinal cord slices and distinguished by the expression of tdTomato protein. At current-clamp mode, rheobase was recorded which refers to the current intensity of 40 ms duration resulting in the first action potential. Besides, the firing pattern was determined by depolarizing pulses of 1 s duration. Unmyelinated primary afferent C fiber evoked excitatory postsynaptic potential (eEPSP) was evoked by electrical stimulation of the dorsal root and judged by stimulation threshold and conduction velocities. Data were collected, digitized, and analyzed by the Axopatch 700B amplifier (Axon Instruments, USA), the Digitizer 1550B, and pCLAMP 10.7 software (Axon Instruments).

### 2.8. Western Blot Analysis

Once the rats were sacrificed, L4-L5 spinal cord tissue was rapidly removed and homogenized in strong RIPA buffer containing 1% protease inhibitors cocktail (Sigma-Aldrich, St Louis, USA) for 20 min and then centrifuged at 12000 rpm/min at 4°C for 15 min to collect supernatant. After protein concentration determined with bicinchoninic acid (BCA) protein assay kit (Cwbiotech, Beijing, China), 40 micrograms of protein samples from different groups were loaded and separated on 10% SDS-PAGE gels and transferred to PVDF membranes (Merck Millipore, Billerica, MA, USA). Blocked with 5% bovine (Beyotime, Shanghai, China) in Tris-buffered saline (pH 7.4) with 0.1% Tween-20 for 2 h at room temperature, the membranes were then incubated overnight at 4°C with primary antibodies. The primary antibodies were rabbit anti *μ*-opioid receptor (1 : 1000, Abcam, ab10275, USA), rabbit anti-*δ* opioid receptor (1 : 1000, Alomone, AOR-014, USA), rabbit anti-cannabinoid receptor 1 (1 : 1000, Cayman Chemical, 101500, USA), rabbit anti-cannabinoid receptor 2 (1 : 1000, Cayman Chemical, 101550, USA), and rabbit anti-GAPDH (1 : 2000, GeneTex, GTX100118, USA). The blots were then incubated with HRP-conjugated secondary antibodies (1 : 8000, Abcam, ab97110, USA) for 2 h. Signals were detected using enhanced chemiluminescent reagent (ECL, Millipore, USA), and the bands were analyzed with the ChemiDoc XRS system (Bio-Rad, Hercules, USA). The quantification of band intensity was carried out using Image software. Band densities were normalized to individual GAPDH internal controls.

### 2.9. ELISA Assays

Rat ELISA kits (Westang, Shanghai, China) of endorphin, enkephalin, MOR, DOR, AEA, 2-AG, CB_1_R, and CB_2_R were used. Rat recombinant cytokine standards and samples of 100 *μ*L were ran in duplicate according to the manufacturer's instructions. The optical density of each well was read at 450 nm.

### 2.10. Intrathecal Catheter Surgery and Drug Administration

To elaborate the difference of analgesic mechanism between single- and combined-acupoint EA, pharmacological experiments were performed. Specific drugs were injected intrathecally and intraperitoneally.

Intrathecal catheter surgery was performed as previously described [34]. After the rats were anesthetized with 1.5% isoflurane in oxygen inhalation, a PE-10 intrathecal catheter was implanted into the intrathecal space of the spinal cord at L4-L6 level. After filling the catheter with sterile endotoxin free PBS, rats were individually housed to protect the catheter from gnawing. Intrathecal injection of 2% lidocaine (10 *μ*L) was performed at 3 days after surgery. A paralysis of the lower limbs occurred within 30 s and recovered within 30 min indicates the success of catheter implantation. Rats without signs of spinal cord damage were applied for experimentation.

The doses were chosen from the previous publication [35], and 10 *μ*g CB_1_R inhibitor AM281 (Sigma-Aldrich, A0980, USA) and 10 *μ*g CB_2_R AM630 (Cayman Chemical, 164178-33-0, USA) were diluted in 15 *μ*L dimethyl sulfoxide (Sigma-Aldrich, 67-68-5, USA) and saline in a ratio of 1 : 1 and then injected through the intrathecal catheter 20 min prior to each EA treatment. Nonselective opioid inhibitor naloxone (1 mg/kg, Tocris Bioscience, UK) was administrated intraperitoneally 2 h prior to each EA treatment as previously reported [36].

#### 2.11. Data Analysis

In the present study, statistical analyses were performed by GraphPad Prism 8.0 (GraphPad Software Inc., La Jolla, USA). All the data were expressed as mean ± SEM. The data of PWMT and numbers of spikes were analyzed by two-way repeated measures analysis of variance (ANOVA) followed by Bonferroni's post hoc analysis. The data of amplitude of eEPSP, rheobase, western blot, and ELISA assay were analyzed by one-way ANOVA and Bonferroni's post hoc analysis. All *P* values were two-sided, and *P* < 0.05 was considered significant.

## 3. Results

### 3.1. The Long-Term Analgesia of Combined-Acupoint EA Is Stronger than That of Single-Acupoint EA

**Figure 1:**
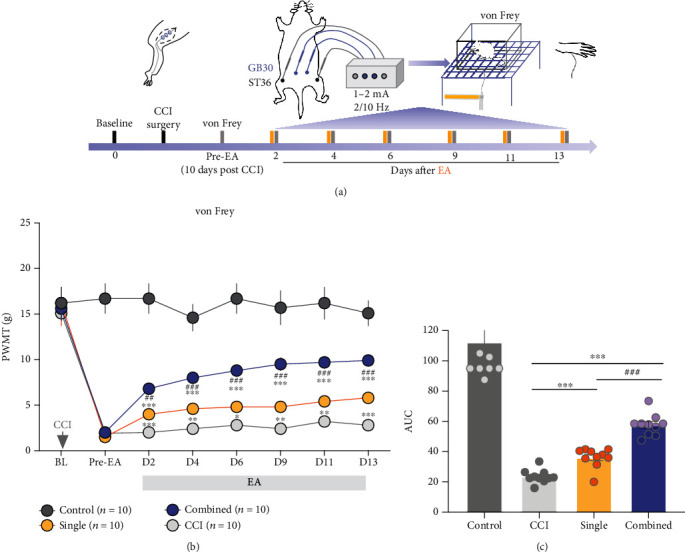
Combined-acupoint EA was more effective than single-acupoint EA in inducing the analgesic effects in CCI rats. (a) Schematic (top) and timeline (bottom) of the CCI model, pain behavior tests (von Frey), and EA stimulation. (b) Time course of CCI injury elicited remarkable mechanical allodynia in the CCI group. Both single-acupoint and combined-acupoint EA alleviated pain, as the paw withdrawal threshold was consistently decreased. The combined-acupoint EA exerted more effective antinociception than single-acupoint EA, and the statistical difference of analgesia between these two groups lasted from day 2 to day 13 of the EA treatment. Two-way ANOVA followed by Bonferroni's multiple comparisons test. (c) Area under the curve of graph b (from “BL” to D13). Student's unpaired *t*-test. All data are mean ± SEM, ^∗^*P* < 0.05, ^∗∗^*P* < 0.01, ^∗∗∗^*P* < 0.001 vs. CCI;^##^*P* < 0.01, ^###^*P* < 0.001 vs. single, *N* = 10 in each group. BL: baseline; CCI: chronic constriction injury; EA: electroacupuncture; AUC: area under curve; PWMT: paw withdrawal mechanical threshold; vs: versus; orange column indicated the EA; gray column indicates the von Frey measurement.

To investigate whether there is any difference in the analgesic effect between EA stimulation at the single and two-combined acupoints, animals were randomly divided into four groups (*n* = 10 per group). Animals in the control group were conducted with sham surgery (the left sciatic nerve was exposed but with no chromic gut sutures tied to it). Animals in the CCI group were conducted with CCI modeling. In the single group, EA treatments were given at bilateral ST36 after CCI modeling. In the combined group, EA stimulation at bilateral ST36 and GB30 was performed after CCI modeling (Figure 1(a)).

As shown in Figure 1(b), the PWMT was dramatically decreased after CCI modeling. EA treatment at both ST36 and ST36 + GB30 significantly increased PWMT at 2, 4, 6, 9, 11, and 13 days after EA (single vs. CCI, *P* < 0.05; combined vs. CCI, *P* < 0.001, Figure 1(b)). Meanwhile, the PWMT of the combined group was higher than that of the single group (single vs. combined, *P* < 0.01), and this difference became statistically significant since the 2 days of EA and maintained until the 13 days. Area under the curve (AUC) of graphs (Figure 1(c)) suggested that the overall effect of alleviating mechanical allodynia was more effective in the combined-acupoint group (single vs. combined, *P* < 0.001). This behavioral result indicates that the long-term analgesic effect of combined-acupoint EA is better than that of single-acupoint EA.

**Figure 2:**
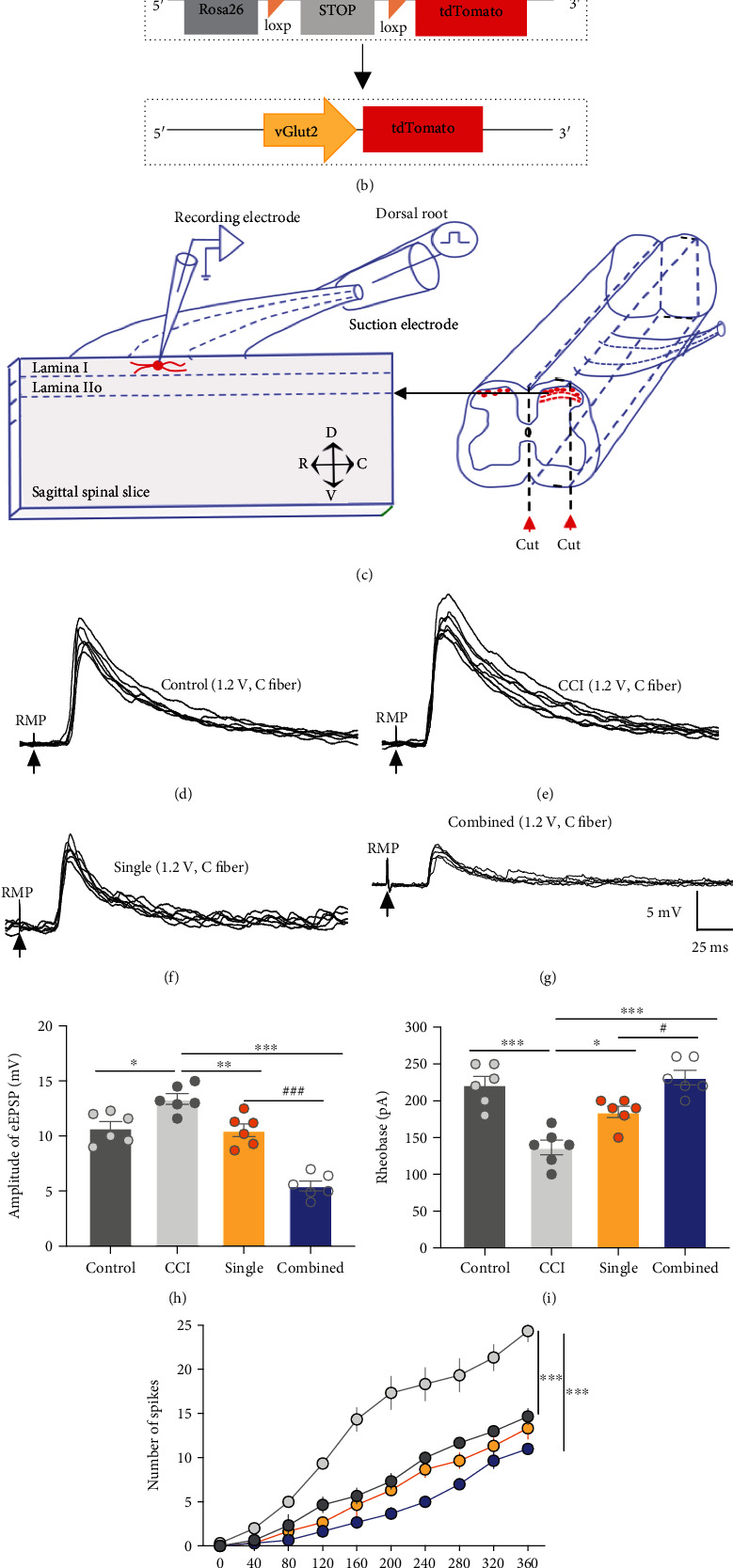
Combined-acupoint EA inhibit the synaptic transmission at the spinal level to a greater extent than Single-acupoint EA in mice. (a) Schematic (top) and timeline (bottom) of the CCI model, pain behavior tests (von Frey), and EA stimulation in control, CCI, combined, and single groups of vGlut2-tdTomato mice. (b) Schematic showing how to mate vGlut2-Cre and Ai9-LSL-tdTomato reporter mice to generate vGlut2-tdTomato mice to specific labeling of vGlut2-positive neurons in spinal dorsal horn. (c) Schematic showing the strategy for electrophysiological whole-cell recording of vGlut2-tdTomato-positive neurons in the lamina I and IIo of sagittal spinal slice attached dorsal root. Representative response of C-fiber evoked EPSP at 1.2 V stimulation of dorsal root in the control group (d) and in the CCI group at 10 days after CCI (e). Representative response of C-fiber evoked EPSP at 1.2 V stimulation of dorsal root in the combined group (f) and single group (g) at 10 days after CCI followed by 6 consecutive days and 1-day off EA treatment. (h) Amplitude of eEPSP recorded in the control, CCI, combined, and single groups. One-way ANOVA and Bonferroni's post hoc analysis. (i) Rheobase of vGlut2-tdTomato neurons in the control, CCI, combined, and single groups. One-way ANOVA and Bonferroni's post hoc analysis. (j) Number of spikes in response to 1000 ms depolarizing current injection of varying amplitude. Two-way repeated measures analysis of variance (ANOVA) followed by Bonferroni's post hoc analysis. (k) Representative tonic firing pattern in response to 1000 ms depolarizing 200 pA current injection. All data are mean ± SEM, ^∗^*P* < 0.05, ^∗∗^*P* < 0.01, ^∗∗∗^*P* < 0.001 vs. CCI; ^##^*P* < 0.01, ^###^*P* < 0.001 vs. single, *N* = 6 in each group. RMP: resting membrane potential; CCI: chronic constriction injury; combined: ST36 + GB30; single: ST36; PWMT: paw withdrawal mechanical threshold; vs: versus; orange column indicated the EA; gray column indicates the von Frey measurement.

In view of synaptic transmission being enhanced in the neuropathic pain conditions and spinal cord mechanism playing an important role in EA analgesia [25, 41], we also investigated changes of synaptic transmission between C fiber and excitatory projection neurons in the lamina I and IIo of vGlut2-tdTomato mice under control, CCI, combined-acupoint EA, and single-acupoint EA conditions (Figures 2(a) and 2(b)). We patched neurons expressing tdTomato in sagittal slices from vGlut2-tdTomato mice (Figure 2(c)). According to the electric strengths for activation of C-fiber and the conduction velocities for C-fiber transmission [33], 1.2 V stimulation of C fiber evoked EPSP was recorded and compared in control, CCI, single, and combined groups. As is shown, the amplitude of the evoked EPSP was higher in CCI mice than in control mice (CCI vs. control, *P* < 0.05, Figures 2(d), 2(e), and 2(h)). Compared with the CCI group, combined and single EA significantly and slightly decreased the amplitude of eEPSP, respectively (combined vs. CCI, *P* < 0.001, Figures 2(e)–2(g)). Meanwhile, compared with single EA, combined EA reduced this amplitude to a greater extent (combined vs. single, *P* < 0.001, Figure 2(h)). Besides, we also detected the excitability of vGlut2-positive neurons in different groups. We found that CCI decreased the rheobase compared to the control group (CCI vs. control, *P* < 0.001, Figure 2(i)). Combined and single EA increased the rheobase compared to the CCI group (combined vs. CCI, *P* < 0.001; single vs. CCI, *P* < 0.05, Figure 2(i)). Tonic firing pattern was recorded in response to prolonged (1000 ms) depolarizing current injections of varying amplitudes (Figure 2(j)). As is shown, CCI increased the action potential firing frequency compared with the control groups under the same current injection amplitude including 200 pA (CCI vs. control, *P* < 0.001, Figures 2(j) and 2(k)). Similarly, action potential firing frequency was significantly inhibited after combined EA (combined vs. CCI, *P* < 0.001, Figures 2(j) and 2(k)). These results suggest that combined-acupoint EA inhibits the synaptic transmission at the spinal level to a greater extent than single-acupoint EA, laying a structural foundation for the behavioral results that combined-acupoint EA significantly alleviated neuropathic pain.

### 3.2. The Stimulation Intensity and Number of Stimulation Sites Are Not the Determinant for the Superior Analgesic Effect of the Combined-Acupoint EA

To further investigate whether the increased number of stimulation sites and the stimulation power in the combined-acupoint EA were the reason for the better analgesic effect, the animals were randomly divided into five groups. The interventions in CCI group, single group, and combined group were the same as those in the last experiment. In the high-intensity group, EA stimulation was given at the maximal tolerable intensity at the acupoint of ST36 (6-8 mA). In the sham-combined group, two nonacupoints (3 mm under ST36 or GB30) were stimulated simultaneously.

**Figure 3:**
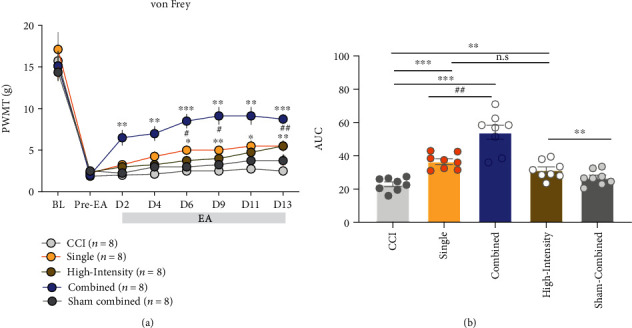
Increasing stimulation intensity or nonacupoint sites for single-acupoint EA did not mimic the analgesic effect of the combined-acupoint EA. (a) The paw withdrawal threshold after single-acupoint EA with high-intensity treatment (6-8 mA) was higher than that of CCI group and not different from that of the single-acupoint group but much lower than that of the combined-acupoint group. EA at two nonacupoints failed to suppress pain. Two-way ANOVA followed by Bonferroni's multiple comparisons test. (b) Area under the curve of graph a (from “BL” to D13). Student's unpaired *t*-test. All data are mean ± SEM, ^∗^*P* < 0.05, ^∗∗^*P* < 0.01, ^∗∗∗^*P* < 0.001 vs. CCI; ^#^*P* < 0.05, ^##^*P* < 0.01 vs. combined; n.s.: not significant; *N* = 8 in each group. BL: baseline; CCI: chronic constriction injury; EA: electroacupuncture; AUC: area under curve; PWMT: paw withdrawal mechanical threshold; vs: versus.

As shown in Figure 3, the PWMT of the single group was significantly higher than that of the CCI group at 6, 9, 11, and 13 days after EA (AUC, single vs. CCI, *P* < 0.001). EA stimulation at ST36 with high intensity also significantly alleviated the mechanical allodynia compared to the CCI group at 13 days after EA (AUC, high-intensity vs. CCI, *P* < 0.01). Besides, the PWMT of the combined group was also significantly higher than that of the CCI group from the 2 to 13 days after EA (AUC, combined vs. CCI, *P* < 0.001). The PWMT of the combined group was also higher than that of the high-intensity group or the single group (AUC, combined vs. high-intensity, *P* < 0.001; combined vs. single, *P* < 0.01). However, there was no significant difference between high-intensity and single group (AUC, high-intensity vs. single, *P* > 0.05), indicating that the increase of stimulation intensity may not be sufficient to enhance the analgesic effect of EA. Meanwhile, there was no statistical difference of PWMT between the sham-combined group and CCI group (AUC, sham-combined vs. CCI, *P* > 0.05), suggesting that increasing number of EA stimulation sites could not strengthen the analgesic effect either.

### 3.3. Both Single- and Combined-Acupoint EA Activated the Endogenous Opioid System

**Figure 4:**
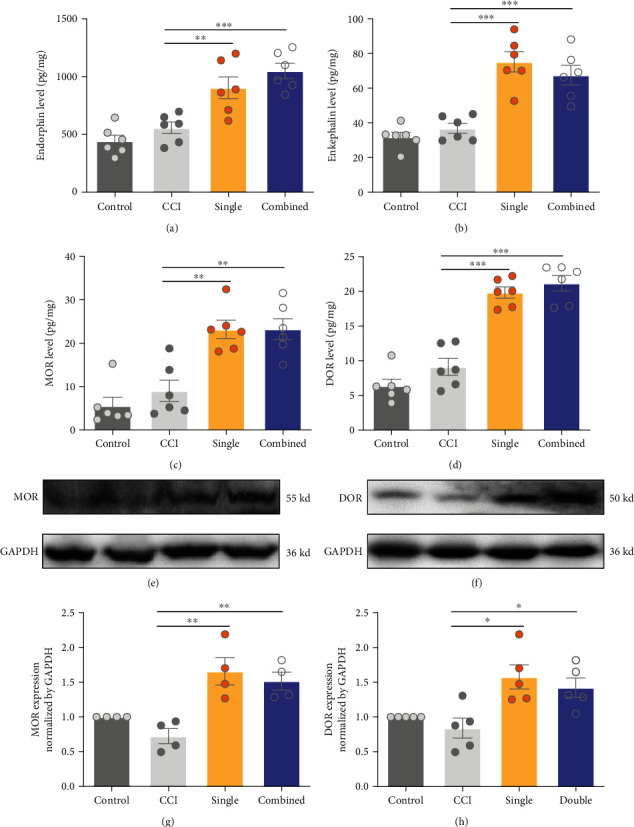
Both single and combined-acupoint EA activated endogenous opioid system. ELISA assay indicated the release of endorphin (a) and enkephalin (b) in both single and combined-acupoint EA groups increased at day 6 after EA treatment. ELISA assay showed that the expression of MOR (c) and DOR (d) was upregulated significantly in the single and combined groups. (e)–(h) The western blot analysis also confirmed the increased expression of MOR and DOR in both single-acupoint and combined-acupoint EA-treated rats. Student's unpaired *t*-test. All data are mean ± SEM, ^∗^*P* < 0.05, ^∗∗^*P* < 0.01, ^∗∗^*P* < 0.01 vs. CCI; *N* = 6 in each group. CCI: chronic constriction injury; EA: electroacupuncture; MOR: *μ*-opioid receptor; DOR: *δ*-opioid receptor.

The endogenous opioid system has been widely reported to participate in the electroacupuncture inhibition of inflammatory and neuropathic pain through peripheral, spinal cord, and supraspinal mechanisms. In inflammatory pain, it has been reported that low frequency EA mainly exerts analgesic effect through met-enkephalin and *β*-endorphin, while high frequency EA relies on dynorphin for analgesia. However, it is unclear whether this specificity still applies to CCI-induced neuropathic pain. So, we compared the endogenous opioids levels and their corresponding receptors between the single-and combined-acupoint EA stimulation. Animals were grouped into control, CCI, single, and combined group randomly, and rats were sacrificed after the EA treatment at 6 days to obtain the spinal cord tissue. As is shown, the endogenous endorphin and enkephalin were increased in both single and combined groups (endorphin: single vs. CCI, *P* < 0.01, combined vs. CCI, *P* < 0.001, Figure 4(a); enkephalin: single vs. CCI, *P* < 0.001, combined vs. CCI, *P* < 0.001, Figure 4(b)). The ELISA assay showed that expressions of MOR and DOR in the spinal cord were significantly elevated in EA treated rats when compared with the CCI group (MOR: single vs. CCI, *P* < 0.01, combined vs. CCI, *P* < 0.001, Figure 4(c); DOR: single vs. CCI, *P* < 0.001, combined vs. CCI, *P* < 0.001, Figure 4(d)), which was also confirmed by the western blot tests (MOR: single vs. CCI, *P* < 0.01, combined vs. CCI, *P* < 0.01, Figures 4(e) and 4(g); DOR: single vs. CCI, *P* < 0.05, combined vs. CCI, *P* < 0.05, Figures 4(f) and 4(h)). These results demonstrated that both single-acupoint and combined-acupoint EA were able to activate the endogenous opioid system. However, the opioids did not contribute to the analgesic discrepancy between single-acupoint and combined-acupoint EA.

### 3.4. Combined-Acupoint Rather than Single-Acupoint EA Activated Endogenous Cannabinoid (eCB) System

**Figure 5:**
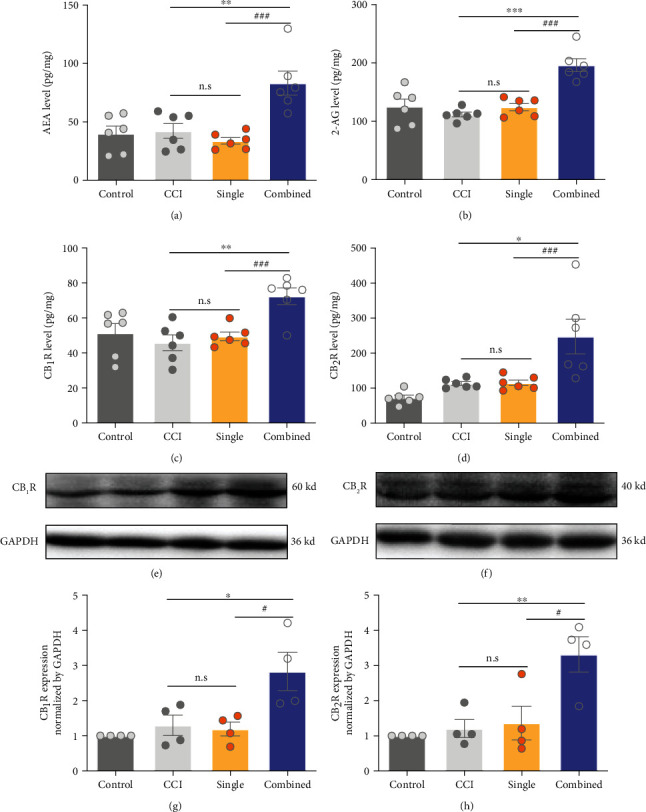
Combined-acupoint rather than single-acupoint EA activated the endogenous cannabinoid (eCB) system. ELISA assay revealed that combined-acupoint EA but not single-acupoint EA increased the release of AEA (a) and 2-AG (b) after 6 days' EA treatment. ELISA assay demonstrated that the expression of CB_1_ (c) and CB_2_ (d) receptor was only upregulated in combined-acupoint EA-treated group. The western blot analysis confirmed combined-acupoint EA but not single-acupoint EA increased the expression of CB_1_ (e, g) and CB_2_ (f, h) in the spinal cord. Student's unpaired *t*-test. All data are mean ± SEM, ^∗^*P* < 0.05, ^∗∗^*P* < 0.01, ^∗∗^*P* < 0.01 vs. combined; ^#^*P* < 0.05, ^##^*P* < 0.01, ^###^*P* < 0.001 vs. combined; n.s.: not significant; *N* = 6 in each group. CCI: chronic constriction injury; EA: electroacupuncture; AEA: anandamide; 2-AG: 2-arachidonoyl glycerol; CB_1_: cannabinoid receptor 1; CB_2_: cannabinoid receptor 2.

The ECS was previously considered as a key factor for EA analgesia through peripheral and supraspinal mechanisms [27]. The ECS consists of endogenous ligands (AEA, 2-AG), cannabinoid receptor (CB_1_R, CB_2_R), synthase, and hydrolase. Hence, we quantified endogenous ligands and cannabinoid receptor level in the groups of single-acupoint and combined-acupoint EA. As illustrated in Figure 5, the combined-acupoint EA increased the release of AEA and 2-AG in comparison with single (AEA, 2-AG: combined vs. single, *P* < 0.001), while the AEA or 2-AG in the single-acupoint EA group did not show significant difference from those in CCI group (AEA, 2-AG: single vs. CCI, *P* > 0.05, Figures 5(a) and 5(b)). Meanwhile, combined-acupoint EA increased the expression of both cannabinoid receptor 1 and 2 protein (ELISA: CB_1_R, CB_2_R, and combined vs. CCI, *P* < 0.001; WB: CB_1_R, CB_2_R, and Combined vs. CCI, *P* < 0.05, Figures 5(c)–5(h)). However, single-acupoint EA did not exert such effect (single vs. CCI, *P* > 0.05, Figure 5). These findings indicated that the specific activation of the spinal cord eCB system might be the cause of the stronger analgesic effect induced by the combined-acupoint EA.

### 3.5. Activation of the Endocannabinoid System Related to the Better Analgesic Effect of the Combined-Acupoint EA

To further elucidate the role of ECS activation in the analgesic effect of combined-acupoint EA, rats with CCI model and intrathecal catheterization were randomly divided into 4 groups: CCI group, single group, combined group, and combined + CBR inhibitor group. All rats received the 2 mg/kg opioid inhibitor naloxone intraperitoneally prior to each EA treatment. At the same time, 10 *μ*g AM281 and 10 *μ*g AM630 were injected through the catheter to the combined + CBR inhibitor group. The PWMT of each rat was then measured at 2, 4, and 6 days after EA treatment.

**Figure 6:**
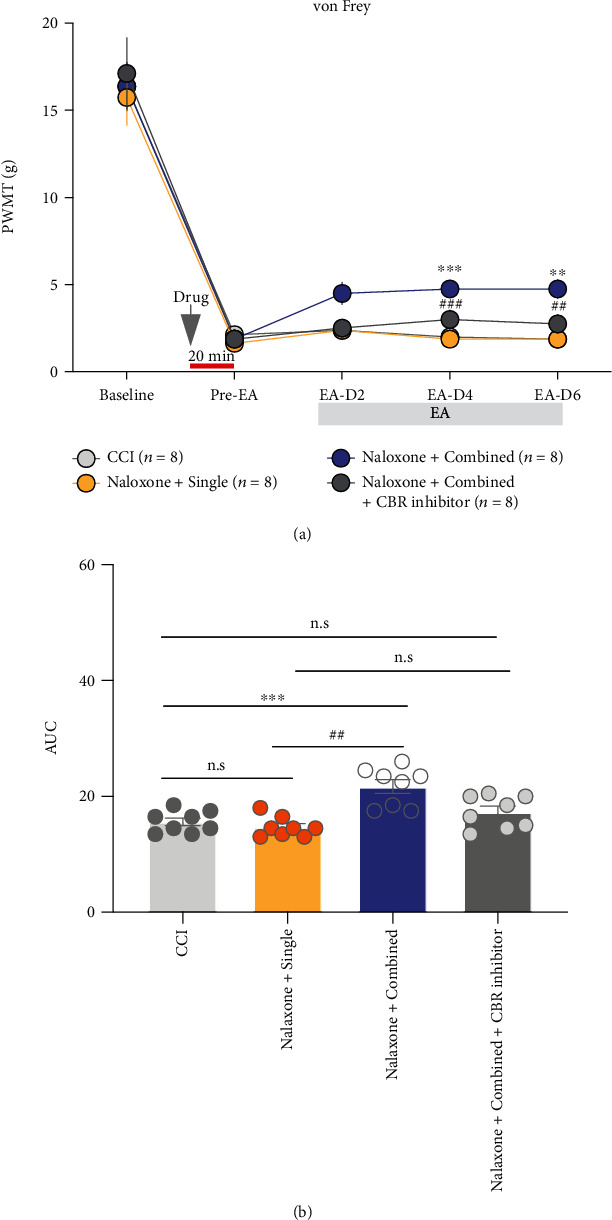
Intrathecal injection of cannabinoid receptor inhibitors blocked the analgesic effect of combined-acupoint EA. (a) Schematic and timeline showing pain behavior tests. All rats were injected with nonselective opioid receptor inhibitor naloxone. Naloxone completely inhibited the antinociceptive effect of single-acupoint EA but just partially reversed the analgesia induced by combined-acupoint EA. Coadministration of naloxone and CBR inhibitors eliminated the analgesic effect of combined-acupoint EA; two-way ANOVA followed by Bonferroni's multiple comparisons test. (b) Area under the curve of graph a (from “BL” to D6). Student's unpaired *t*-test. All data are mean ± SEM, ^∗^*P* < 0.05, ^∗∗^*P* < 0.01, ^∗∗^*P* < 0.01 vs. CCI; ^##^*P* < 0.01, ^###^*P* < 0.001 vs. naloxone + single; n.s.: not significant; *N* = 8 in each group. CCI: chronic constriction injury; EA: electroacupuncture; PWMT: paw withdrawal mechanical threshold; CBR: cannabinoid receptor.

As illustrated in Figure 6, the PWMT of rats in the combined group were significantly higher than that of CCI or single group at 4 (naloxone + combined vs. CCI, *P* < 0.001; naloxone + combined vs. naloxone + single, *P* < 0.001) and 6 days (naloxone + combined vs. CCI, *P* < 0.01; naloxone + combined vs. naloxone + single, *P* < 0.01). However, the PWMT in the single group was not different from that in the CCI group (naloxone + single vs. CCI, *P* > 0.05). When treated with cannabinoid receptor inhibitors, the pain alleviation effect of the combined-acupoint EA was blocked, as the PWMT of combined + CBR inhibitor was not different from CCI or single group (naloxone + combined + CBR inhibitor vs. CCI, *P* > 0.05; naloxone + combined + CBR inhibitor vs. naloxone + single + CBR inhibitor, *P* > 0.05, Figure 6(a)). The AUC results also showed that blocking the opioid receptor can completely block the analgesia effect of single EA (naloxone + single vs. CCI, *P* > 0.05); however, only simultaneously blocking both opioid and cannabinoid receptors can block the analgesia effect of combined EA (Figure 6(b)). These behavioral results illustrated that the endogenous opioid system was involved in the analgesia of both single-and combined-acupoint EA, while the eCB system was related to the superiority of analgesia induced by the EA stimulation at ST36 and GB30.

### 3.6. EA Stimulation at Single-Acupoint GB30 Was Unable to Provide a Superior Analgesic Effect than ST36 in the ECS Independent Manner

**Figure 7:**
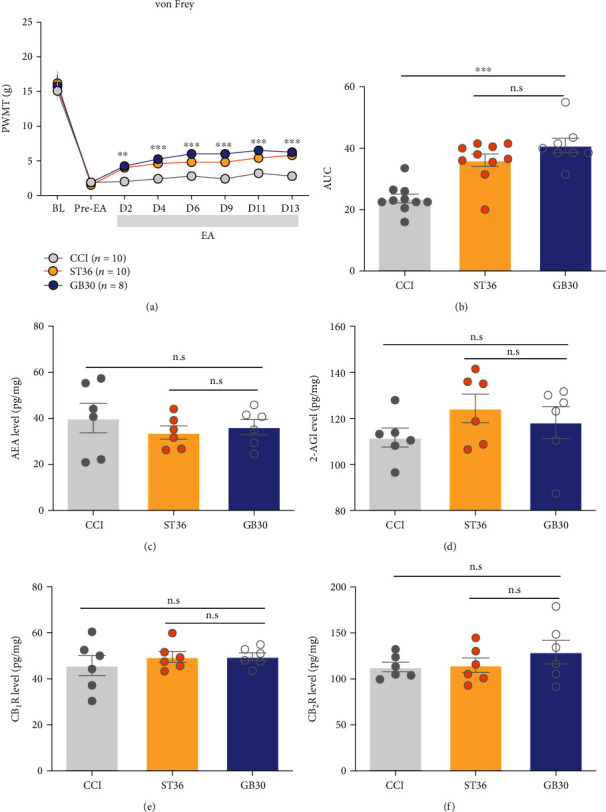
EA stimulation at single-acupoint GB30 was unable to provide a superior analgesic effect than ST36 in the ECS independent manner. (a) EA stimulation at GB30 provided a comparable analgesic effect to EA stimulation at ST36; two-way ANOVA followed by Bonferroni's multiple comparisons test. (b) Area under the curve of graph a (from “BL” to D13). Student's unpaired *t*-test. EA stimulation at GB30 did not elevate the AEA (c), 2-AG (d), CB_1_R (e), and CB_2_R (f) which activate the endocannabinoid system. All data are mean ± SEM, ^∗∗^*P* < 0.01, ^∗∗∗^*P* < 0.001 vs. CCI; n.s.: not significant; *N* = 6 in each group. CCI: chronic constriction injury; EA: electroacupuncture; eCB: endocannabinoid; AEA: anandamide; 2-AG: 2-arachidonoyl glycerol; CB_1_: cannabinoid receptor 1; CB_2_: cannabinoid receptor 2; ST36: Zusanli acupoint; GB30: Huantiao acupoint.

To further explore the mechanisms of ECS-mediated analgesia superiority of combined-acupoint EA, experiments about EA stimulation at GB30 alone were performed. Rats were randomly divided into CCI, ST36, and GB30 groups, which the latter two groups received the EA stimulation at ST36 and GB30, respectively. As is shown, EA stimulation at GB30 significantly alleviated the mechanical allodynia compared with the CCI group from the 2 days after EA stimulation (PWMT: GB30 vs. CCI, *P* < 0.01, Figure 7(a)), paralleled by the difference of overall effect reflected by the AUC (GB30 vs. CCI, *P* < 0.001, Figure 7(b)). However, compared with the single-acupoint stimulation at ST36, EA stimulation at GB30 alone did not provide a superior analgesic effect on the mechanical allodynia (PWMT: GB30 vs. ST36, *P* > 0.05, Figure 7(a); AUC: GB30 vs. ST36, *P* > 0.05, Figure 7(b)). Besides, EA stimulation at GB30 did not alter the content of AEA, 2-AG, CB_1_, and CB_2_ receptor compared with CCI (GB30 vs. CCI, *P* > 0.05, Figures 7(c)–7(f)) or ST36 (GB30 vs. ST36, *P* > 0.05, Figures 7(c)–7(f)) group, excluding the possibility that GB30 acupoint itself determined the analgesia superiority of combined-acupoint EA mediated by the ECS.

## 4. Discussion

The present study revealed that the analgesic effect of EA stimulation at combined acupoints ST36 and GB30 was stronger than that of EA stimulation at ST36 alone in the CCI animal model. Combined-acupoint EA significantly inhibited the synaptic transmission of pain information in the spinal dorsal horn compared to the single-acupoint EA to a greater extent. The increase of the stimulating intensity or number of stimulation sites did not enhance the analgesic effect of single-acupoint EA. Intraperitoneal naloxone injection could reverse the pain alleviation induced by single-acupoint EA, but not the combined-acupoint EA, unless inhibiting endogenous cannabinoid receptors. These findings provided evidence of the advantage of acupoints combination EA in the treatment of neuropathic pain and shed light on the underlying mechanism of acupuncture induced analgesia.

The current results demonstrated that combined-acupoint EA is more effective than single-acupoint EA in pain alleviation, which the PWMT of the combined group being significantly higher than the single group since the 2 days of EA treatment. This result corresponds to a previous study, in which combined-acupoint EA was demonstrated to provide stronger antinociception than single-acupoint EA did in an incisional pain model [37, 38]. Besides, the enhancement of synaptic transmission in the spinal cord is one of the mechanisms of neuropathic pain [23, 39]. Although related studies have reported that EA alleviates neuropathic pain by modulating the long-term synaptic plasticity in the spinal dorsal horn through field potential recording [40], they did not detect whether this effect is still applicable to explain the superiority of combined-acupoint stimulation. So, we further found that combined-acupoint EA produced superior analgesia through inhibiting the synaptic transmission between the nociceptive primary afferent and excitatory projection neurons using the whole-cell recording. Meanwhile, we also found higher-intensity stimulation, and the nonacupoint site stimulation failed to provide stronger analgesic effect, which indicated the stimulation at acupoint but not the electrical stimulus itself is responsible for achieving the better analgesic effect of combined-acupoint EA. However, whether EA induced analgesia depends on the power of the electrical stimulus is still controversial. Some studies reported that EA with higher intensity provided more powerful analgesia in healthy volunteers [41, 42]; however, some found mild EA was sufficient to produce analgesia in CFA induced inflammatory pain. The theory of “acupoint sensitization” may help to explain the paradoxical results. In this theory, acupoints are in “silent” state under physiological condition but are activated under pathological condition, which makes them more sensitive to external force, heat, light, electricity, and other stimuli [43].

The involvement of opioid substances in mediating acupuncture-induced analgesia was firstly demonstrated in the 1970s [44]. Acupuncture has been proved to activate all the three subfamilies of the endogenous opioid peptide, endorphin, enkephalin, and dynorphin. Low-frequency EA (2 Hz) mainly causes the release of endorphin and enkephalin, and high-frequency EA (100 Hz) dominates the activation of the dynorphin system in the inflammatory pain [26, 45]. In the present study, we found that the dense-disperse mode at 2/10 Hz in the CCI-induced neuropathic pain model used for EA stimulation also induced the release of endorphin and enkephalin and upregulated their receptors MOR and DOR, followed by the contents of dynorphin being not elevated by EA treatment under the low-frequency, which is similar with inflammatory pain. This indicates that there may be no difference between the endogenous opioid system in regulating inflammatory pain and CCI-induced chronic pain.

The ECS is also a key factor in analgesia induced by EA [46]. Multiple studies have shown that acupuncture was able to activate CB1 receptor in the central nervous system [47, 48] and CB2 receptor in the peripheral [49, 50] to produce an analgesic effect. However, whether the ECS in the spinal cord participates in EA-induced analgesia remains unclear. In the current study, we found that EA at bilateral ST36 and GB30 rather than ST36 alone induced the release of both AEA and 2-AG and upregulated both CB_1_ and CB_2_ receptors in the spinal cord in the CCI animal model. There could be a possibility that the acupoint of GB30 was responsible for the activation of the ECS. However, our results showed that EA stimulation at GB30 alone neither provided a superior analgesic effect to EA treatment at ST36 nor altered the content of AEA, 2-AG, CB_1_, or CB_2_ receptor compared with the CCI group. This result suggested that the activation of ECS and the superior antinociception of combined-acupoint EA were not due to the simple combination of two acupoints' function but probably originated from a complex synergy effect of stimulating these two acupoints during neuropathic pain pathology. Up to now, most studies which reported the combined-acupoint EA could suppress pain via regulating ECS were done in the CFA model or knee osteoarthritis model in the supraspinal and peripheral level [51–54]. However, there were some studies showing single-acupoint EA could also activate the ECS to suppress acute pain elicited by heat stimuli [55, 56]. This inconsistency is probably caused by the heterogeneous mechanism of pain models. As explained by the theory of traditional Chinese medicine, single-acupoint acupuncture is mainly used to treat simple and emergency symptoms, while combined-acupoint acupuncture is used to cure complex and chronic diseases [56, 57]. The CCI neuropathic pain model we used in the current study is a kind of chronic pain model, in which no study ever reported the involvement of ECS activation in the single-acupoint EA. However, whether single-acupoint EA may have a transient regulatory effect on the ECS in the early stage after CCI modeling needs to be confirmed in further research. Moreover, it is generally known that ECS participates in the regulation of neuropathic pain at the spinal cord [58]. So, it is also very important to further study the role of ECS in modulating the inhibition of synaptic transmission in the spinal dorsal horn by different EA schemes. Certainly, we admit that there could be the influence of anatomical and physiological distinctions between rats and mice on the electroacupuncture. However, in clinical, patients receive the electroacupuncture normally in awake state. In order to perform the electroacupuncture to rodents without anesthesia, we used rats for most our studies, because rats could be easily immobilized in the homemade fixing platform without anesthesia, but it is harder to deliver electroacupuncture in mice at awake state. However, in the electrophysiological experiment, to specifically record the vGlut2-positive neurons, the vGlut2-tdTomato transgenic mice were used in respect of lacking of corresponding transgenic rats. Besides, to thoroughly elucidate how did ECS mediate analgesia discrepancy between the single and combined-acupoint EA, further exploration of the molecular and circuitry involving ECS in the spinal cord is needed.

## 5. Conclusion

In summary, the present study showed that the analgesic effect induced by combined EA stimulation at ST36 and GB30 is stronger than that induced by EA stimulation at ST36 alone in the CCI-induced neuropathic pain animal model. This superiority of analgesia is closely related to the specific activation of the ECS in the spinal cord by the combination of ST36 and GB30 stimulation, which provides a structural and functional basis for emphasizing electroacupuncture as an important complementary treatment.

## Data Availability

The data supporting the findings of this study is available from the corresponding authors.
